# WNT-pathway medulloblastoma: what constitutes low-risk and how low can one go?

**DOI:** 10.18632/oncotarget.28360

**Published:** 2023-02-07

**Authors:** Shakthivel Mani, Abhishek Chatterjee, Archya Dasgupta, Neelam Shirsat, Sridhar Epari, Girish Chinnaswamy, Tejpal Gupta

**Affiliations:** ^1^Department of Radiation Oncology, ACTREC/TMH, Tata Memorial Centre, Homi Bhabha National Institute (HBNI), Mumbai, Maharashtra 410210, India; ^2^Department of Neuro-Oncology Laboratory, ACTREC/TMH, Tata Memorial Centre, Homi Bhabha National Institute (HBNI), Mumbai, Maharashtra 410210, India; ^3^Department of Pathology, ACTREC/TMH, Tata Memorial Centre, Homi Bhabha National Institute (HBNI), Mumbai, Maharashtra 410210, India; ^4^Department of Pediatric Oncology, ACTREC/TMH, Tata Memorial Centre, Homi Bhabha National Institute (HBNI), Mumbai, Maharashtra 410210, India

**Keywords:** de-intensification, medulloblastoma, molecular, survival, toxicity

## Abstract

Novel biological insights have established that medulloblastoma is a heterogenous disease comprising four broad molecular subgroups - WNT, SHH, Group 3, and Group 4 respectively, resulting in the incorporation of molecular/genetic information in 5th edition of WHO classification and contemporary risk-stratification. Concerns regarding therapy-related late toxicity in long-term survivors have led to systematic attempts at treatment de-intensification in good-risk medulloblastoma. Given the excellent survival (>90%) of WNT-pathway medulloblastoma, prospective clinical trials have focused on optimization of therapy to balance survival versus quality of survival. The currently accepted definition of low-risk WNT-pathway medulloblastoma includes children <16 years of age with residual tumour <1.5 cm^2^ and no evidence of metastases. This systematically excludes adolescents and young adults who have been perceived to have worse outcomes. We have previously reported long-term survival of our adolescent and young adult cohort that were largely comparable to childhood medulloblastoma. We now report on molecularly characterized WNT-subgroup patients treated between 2004–2020 with risk-stratified multi-modality therapy to identify differences between childhood (<15 years) versus adolescent and young adults (>15 years). Despite modest differences in disease status at presentation and treatment modality, there were no significant differences in patterns of failure or survival between childhood versus adolescent and young adult WNT-pathway medulloblastoma. Two de-intensification trials in low-risk WNT-pathway medulloblastoma – first testing omission of upfront craniospinal irradiation and second a primary chemotherapy approach after surgery – had to be terminated prematurely due to unacceptably high relapse rates suggesting that craniospinal irradiation remains an integral component of treatment. The presence of *TP53* mutations and *OTX2* gains have recently been reported as independent negative prognostic factors in a multi-institutional cohort of WNT-pathway medulloblastoma raising questions on eligibility of such patients for de-escalation trials. The definition of low-risk WNT-pathway medulloblastoma may need to be refined in light of recent clinical data and newer biological information.

## INTRODUCTION

Medulloblastoma (MB) is the most common malignant central nervous system (CNS) neoplasm in children comprising 20–25% of all primary brain tumors [1]. The current standard-of-care for non-infantile MB comprises maximal safe resection followed by conventional risk-stratified post-operative radiotherapy (RT) and 6–8 cycles of adjuvant systemic chemotherapy [2, 3]. Given the high propensity of neuraxial spread via cerebrospinal fluid (CSF) pathways, craniospinal irradiation (CSI) to a dose of 23.4–36 Gy plus posterior fossa/tumor-bed boost (18–30.6 Gy) for total primary-site dose of 54–55 Gy remains the cornerstone of adjuvant RT in medulloblastoma [4–6]. Traditionally, children over the age of 3 years with no or small post-operative residual tumor (<1.5 cm^2^) and no evidence of leptomeningeal metastases (M0) were classified as average-risk disease [5] with >80% long-term survival [4–6]. Conversely, presence of one or more high-risk features [5] defined as age <3 years, residual disease ≥1.5 cm^2^, or metastases (M+) resulted in much worse 5-year survival (30–60%) despite aggressive multi-modality therapy [7].

Novel biological insights have vastly improved our fundamental understanding of pediatric brain tumors with potential to transform therapy [2, 3]. It is now well established that MB is a heterogenous disease [8–10] comprising four broad molecular subgroups - wingless (WNT), sonic hedgehog (SHH), Group 3, and Group 4 respectively with unique developmental origins, distinct molecular pathways, diverse phenotypes, and varying clinical behaviour prompting the inclusion of genetic based classification in the 5th edition of the World Health Organization (WHO) classification of CNS tumors [11]. The risk-stratification schema [5] which was hitherto based entirely on clinico-radiological grounds has been refined by incorporating molecular/genetic information into low-risk, standard-risk, high-risk, and very high-risk with expected 5-year survival of >90%, 75–90%, 50–75%, and <50% respectively in the molecular era [12].

### Balance between survival and quality of survival

Contemporary aggressive multi-modality treatment provides excellent long-term survival particularly in WNT-MB with 5-year survival exceeding 90% [12, 13]. However, this results in significant toxicity in long-term survivors, especially children who are more vulnerable and susceptible to RT dose and volume-dependent late morbidity such as neuro-cognitive deficits, endocrinopathies, sensori-neural hearing impairment, cerebro-vascular accidents, cardio-pulmonary toxicity and second malignant neoplasms [14, 15]. An optimal balance needs to be reached between survival versus therapy-related toxicity and its resultant impact upon quality of survival through conduct of subgroup-specific prospective clinical trials [2, 16] of treatment de-intensification in MB. The currently accepted definition of low-risk WNT-MB [5] includes children <16 years of age with residual tumour <1.5 cm^2^ and no evidence of metastases. This systematically excludes adolescents and young adults (AYAs) who have been perceived to have worse outcomes compared to their childhood counterparts. An analysis of the Surveillance, Epidemiology, and End Results (SEER) database [17] from 1992–2013 reported comparable 2-year, 5-year, and 10-year survival between childhood (*n* = 616) and adult MB (*n* = 349). We have also previously reported long-term survival outcomes of our AYA-MB cohort [18] that were largely comparable to childhood MB. We now reviewed our molecularly characterized WNT-MB cohort based on differential expression of 12 protein-coding genes and 9 microRNAs [19] treated with risk-stratified multi-modality therapy between 2004-2020 to identify differences if any between childhood (<15 years) and AYA (≥15 years) WNT-MB in terms of presentation, treatment modality, patterns of failure, and survival. Patient, disease, and treatment characteristics were compared using chi-square test or Fishers’ exact test as appropriate. Time-to-event outcomes were analysed using Kaplan-Meier method and compared with log-rank test. Any *p*-value <0.05 was considered as statistically significant. All analysis was done on SPSS version 24.0 and RStudio version 4.03.

**Figure 1:**
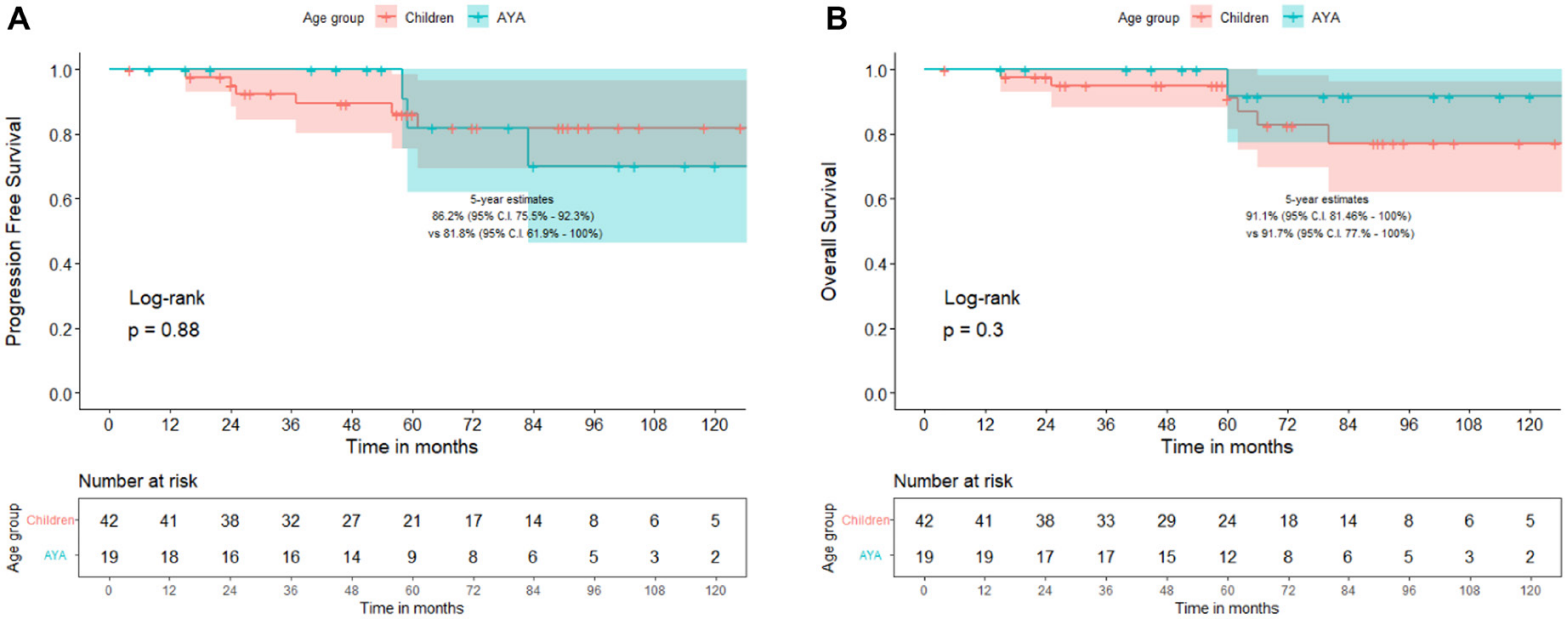
Comparison of progression-free survival (**A**) and overall survival (**B**) between children (<15 years) versus adolescents and young adults (AYAs ≥15 years) with WNT-pathway medulloblastoma.

During this time-period, a total of 67 patients - 44 children (<15 years) and 23 AYAs (≥15 years) were diagnosed with WNT-MB at our institute. Patient, disease, treatment characteristics and patterns of failure are summarized in Table 1. Five children had metastases at presentation compared to none from AYA cohort. Classic MB was predominant histological subtype in children whereas MB - not otherwise specified was commonly seen in AYA cohort. Children with WNT-MB were more likely to have received adjuvant systemic chemotherapy. Conversely, AYA WNT-MB were more likely to have received higher CSI doses. There were no significant differences in patterns of failure between childhood and AYA WNT-MB cohorts (Table 1). At a median follow-up of 72 months (inter-quartile range 51–101 months), 5-year Kaplan-Meier estimates of progression-free survival (PFS) with 95% confidence interval (CI) were 86.2% (95% CI: 75.5–92.3%) for childhood WNT-MB compared to 81.8% (95% CI: 61.9–100%) for the AYA cohort (*p* = 0.80, Figure 1A). Similarly, there were no significant differences in 5-year overall survival (OS) with Kaplan-Meier estimates of 91.1% (95% CI: 81.5–100%) and 91.7% (95% CI:77–100%) respectively (*p* = 0.30, Figure 1B).

**Table 1:** Patient, disease, and treatment characteristics of the study cohort

Characteristics	Childhood WNT-MB	AYA WNT-MB	*p*-value
**Sex**	(*n* = 44)	(*n* = 23)	0.54
Male	30 (68%)	19 (82%)	
Female	14 (32%)	4 (18%)	
**Metastatic status at presentation**	(*n* = 42)	(*n* = 20)	0.12
No metastases (M0)	37 (88%)	20 (100%)	
Presence of metastases (M+)	5 (12%)	0 (0%)	
**Residual disease**	(*n* = 40)	(*n* = 17)	0.45
<1.5 cm^2^	32 (80%)	15 (88%)	
≥1.5 cm^2^	8 (20%)	2 (12%)	
**Histological subtype**	(*n* = 44)	(*n* = 23)	**0.009**
Medulloblastoma - NOS	12 (27%)	10 (43%)	
Classic	32 (73%)	9 (39%)	
Desmoplastic	0 (0%)	3 (13%)	
Large-cell/anaplastic	0 (0%)	1 (5%)	
**Risk-stratification**	(*n* = 40)	(*n* = 15)	0.51
Average-risk	27 (67.5%)	12 (80%)	
High-risk	13 (32.5%)	3 (20%)	
**Adjuvant chemotherapy**	(*n* = 38)	(*n* = 16)	**0.04**
Yes	31 (81%)	8 (50%)	
No	7 (19%)	8 (50%)	
**Dose of craniospinal irradiation**	(*n* = 38)	(*n* = 16)	0.09
23.4 Gy	19 (50%)	4 (25%)	
35 Gy	19 (50%)	12 (75%)	
**Patterns of failure**	(*n* = 5)	(*n* = 3)	0.16
Tumor-bed/posterior fossa	2 (40%)	0 (0%)	
Leptomeningeal dissemination	2 (40%)	0 (0%)	
Combined local + leptomeningeal	1 (20%)	2 (66.7%)	
Extra-neuraxial metastases	0 (0%)	1 (33.3%)	

All statistically significant *p*-values are highlighted in bold. Abbreviations: WNT: wingless; MB: medulloblastoma; AYA: adolescent and young adult; NOS: not otherwise specified.

Our results are in accordance with previously published reports. Nobre et al. [20] reported on relapses from a retrospective multi-institutional clinically annotated cohort of WNT-MB (*n* = 93). Fifteen patients with relapse were identified, 12 in metastatic compartment including one with extra-neuraxial metastases and 3 in the surgical cavity. Lower cumulative dose of cyclophosphamide/ifosfamide (<12 mg/m^2^) during maintenance chemotherapy (*p* = 0.032) and male gender (*p* = 0.033) were associated with significantly increased risk of relapse. Age at diagnosis, extent of resection, metastases, CSI dose, and additional molecular/genetic alterations did not influence the risk of relapse. More recently, 5-year PFS and OS of 100% has been reported in WNT-MB in conventionally classified average-risk (*n* = 46) as well as high-risk disease (*n* = 7) from a prospective cohort study of risk-adapted therapy (SJMB-03) [21]. Another prospective trial [22] that included patients from 3–21 years of age with average-risk MB reported 5-year PFS and OS of 93.3% and 95.5% respectively in the WNT-subgroup (*n* = 64) confirming excellent outcomes regardless of risk-stratification and age. In accordance with above retrospective and prospective clinical data, we believe that age alone should not preclude WNT-MB patients from participation in prospective clinical trials testing treatment de-intensification.

### How low can one go

Attempts at treatment de-escalation in non-metastatic childhood MB are not new and have been attempted systematically since the last 40 years. The first successful de-intensification was the reduction from full-dose CSI (36 Gy) to reduced-dose CSI (23.4 Gy) with addition of adjuvant systemic chemotherapy in children with average-risk MB [4] which was subsequently adopted as the standard dose of CSI in children with average-risk disease. Results of subsequent attempts at de-escalation in non-metastatic childhood MB have been somewhat mixed. However, caution is warranted during any de-escalation of RT even in favorable biology disease. Replacing CSI with high-dose chemotherapy using thiotepa-based conditioning and autologous stem cell rescue has been attempted in older children (up to 10 years) similar to infant MB but with limited success [23]. The acute and late toxicity of such an approach also needs to be carefully weighed against the anticipated late morbidity of lower dose (18–23.4 Gy) CSI in young children (3–10 years). Previously published data show that deferral of RT [24] or reduction of CSI dose [22] in younger children (aged between 3 to <8 years) with molecularly unselected medulloblastoma results in inferior survival, suggesting that early RT, particularly CSI (in appropriate doses) remains an integral component of treatment. We have previously reported unacceptably high risk of neuraxial failure with omission of upfront CSI in children with rigorous-defined low-risk WNT-MB [25]. Similar results were reported with primary chemotherapy approach only after surgery [26, 27] leading to early termination of both these studies. Although the results of both these trials of treatment de-intensification were disappointing, the search for optimal balance between quality of life and survival needs to continue across all subgroups of MB including WNT [28]. The optimal dose of CSI in low-risk WNT-MB remains an area of active investigation in ongoing prospective clinical trials [2, 16] such as the SJMB-12 (NCT01878617), COG ACNS1422 (NCT02724579), PNET-5 (NCT02066220) and FOR-WNT 2 (NCT04474964). The SJMB-12 study evaluating 15 Gy CSI plus boost RT for total primary site dose of with 50 Gy primary-site dose followed by 4 cycles of cisplatin, cyclophosphamide, and vincristine in low-risk WNT stratum has recently completed accrual and may emerge as the next standard-of-care for low-risk disease if such an approach is associated with >90% long-term survival. Subsequently efforts to further reduce CSI dose to 12 Gy may need to be tested in the future.

Various molecular markers and associated signalling pathways involved in metastases from MB have been described [29]. However, given >95% survival in adequately and appropriately treated patients of WNT-MB, prognostic factors affecting outcomes, patterns of failure, and drivers of metastatic dissemination are not well understood in this subgroup. In a recent large multi-institutional cohort of 191 patients of WNT-MB, presence of *TP53* mutations or *OTX2* gains emerged as independent poor prognostic markers [30] raising questions on eligibility of such patients for de-escalation trials. Second generation molecular subgrouping [31] has identified multiple subgroups within each broad molecular subgroup which could impact the conduct of subgroup-specific clinical trials in the future.

## CONCLUSIONS

Concerns regarding therapy-related late toxicity have prompted systematic attempts at treatment de-intensification in good-risk MB over the last four decades. However, results of prior studies should be used to inform and guide controlled de-intensification of therapy even in low-risk and favourable biology disease. The definition of low-risk WNT-MB may need to be further refined in light of recent clinical data and newer biological information.
